# Supplementary figures and images for: Effects of bortezomib on intracellular antioxidant and apoptosis in HepG2cells
Source: PeerJ. 2025 Apr 28;13:e19235. doi: 10.7717/peerj.19235 (PMC12045286; doi:10.7717/peerj.19235)

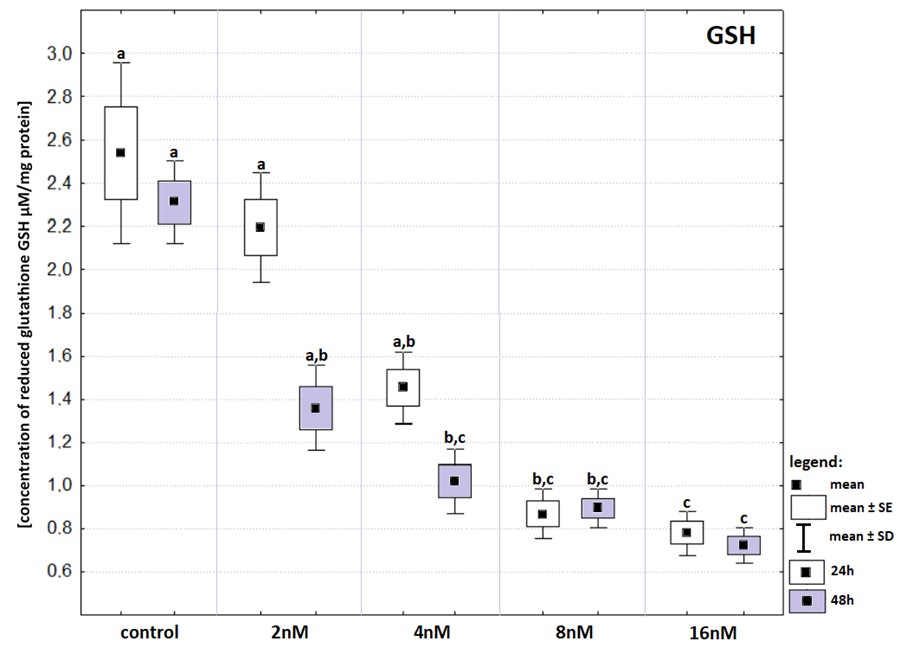

Supplement: Supplemental Information 1 — Values are presented as mean ±SE and mean ±SD. Letters indicate groups that differ significantly from each other (p < 0.05, Kruskal–Wallis test and Dunn’s test as a post hoc); (a) significantly different from values marked with letters b and c; (ab) significantly different from values marked with a letter c; (bc) significantly different from values marked with letter a. [file peerj-13-19235-s001.jpg]

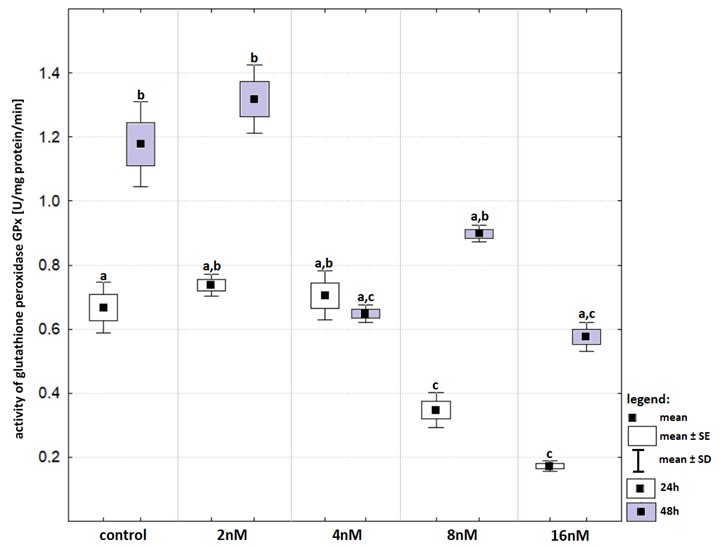

Supplement: Supplemental Information 2 — Activity of glutathione peroxidase (in relation to that recorded in control groups) in cell lines exposed to bortezomib at concentrations of 2 nM, 4 nM, 8 nM and 16 nM for 24-hours (white boxes) and 48-hours (grey boxes). Values are presented as mean ±SE and mean ±SD. Letters indicate groups that differ significantly from each other (p < 0.05, Kruskal–Wallis test and Dunn’s test as a post hoc); (a) significantly different from values marked with letters b and c; (b) significantly different from values marked with letters a and c; (ab) significantly different from values marked with letter c; (ac) significantly different from values marked with letter b. [file peerj-13-19235-s002.jpg]

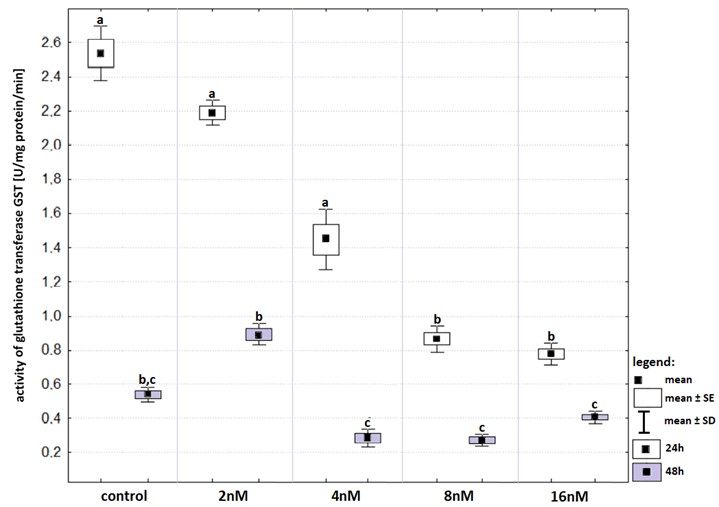

Supplement: Supplemental Information 3 — Values are presented as mean ±SE and mean ±SD. Letters indicate groups that differ significantly from each other (p < 0.05, Kruskal–Wallis test and Dunn’s test as a post hoc); (a) significantly different from values marked with letters b and c; (b) significantly different from values marked with letters a and c; (c) significantly different from values marked with letters a and b; (bc) significantly different from values marked with letter a. [file peerj-13-19235-s003.jpg]

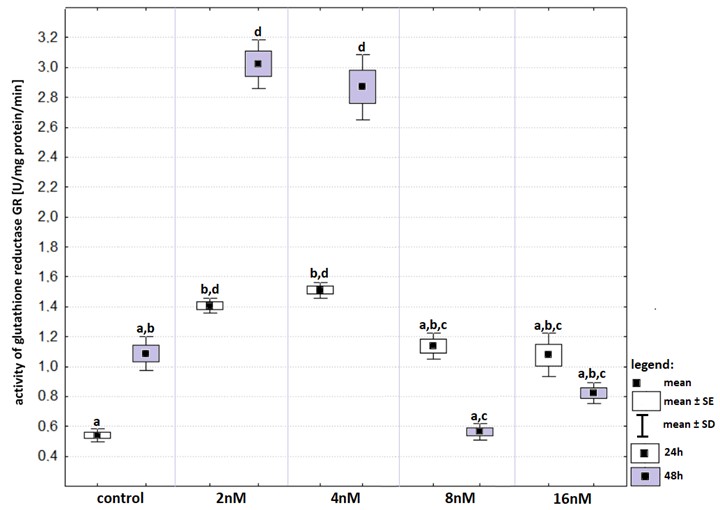

Supplement: Supplemental Information 4 — Values are presented as mean ±SE and mean ±SD. Letters indicate groups that differ significantly from each other (p < 0.05, Kruskal–Wallis test and Dunn’s test as a post hoc); (a) significantly different from values marked with letters b, c and d; (ab) significantly different from values marked with letters c and d; (bd) significantly different from values marked with letters a and c; (d) significantly different from values marked with letters a, b and c; (abc) significantly different from values marked with letter a; (ac) significantly different from values marked with letters b and d. [file peerj-13-19235-s004.jpg]

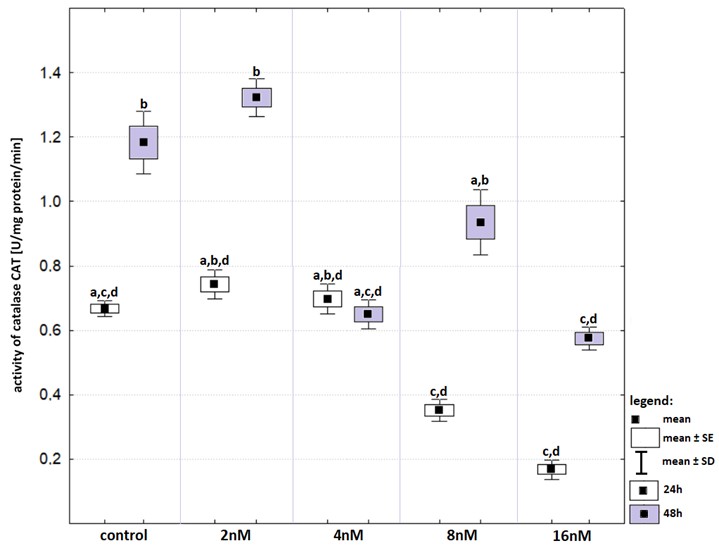

Supplement: Supplemental Information 5 — Values are presented as mean ±SE and mean ±SD. Letters indicate groups that differ significantly from each other (p < 0.05, Kruskal–Wallis test and Dunn’s test as a post hoc); (b) significantly different from values marked with letters a, c and d; ab-significantly different from values marked with letters c and d; (cd) significantly different from values marked with letters a and b; (acd) significantly different from values marked with letter b; (abd) significantly different from values marked with letter c. [file peerj-13-19235-s005.jpg]

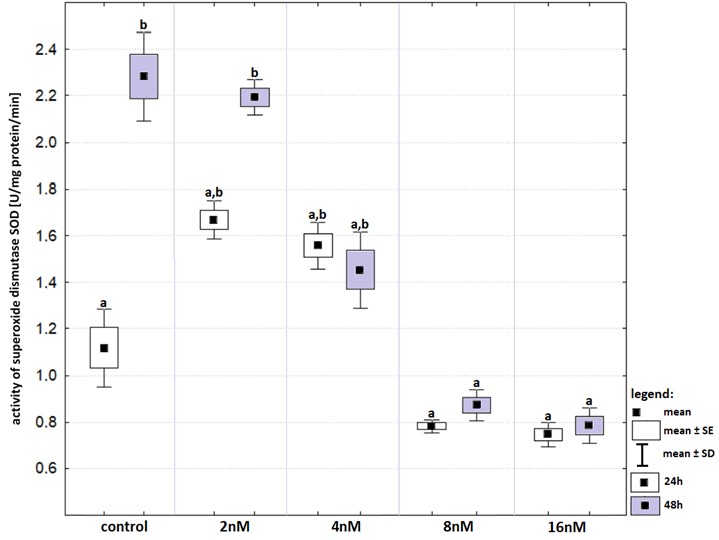

Supplement: Supplemental Information 6 — Values are presented as mean ±SE and mean ±SD. Letters indicate groups that differ significantly from each other (p < 0.05, Kruskal–Wallis test and Dunn’s test as a post hoc); (a) significantly different from values marked with letter a; (b) significantly different from values marked with letter a; (ab) no significantly different from values marked with letters a and b. [file peerj-13-19235-s006.jpg]

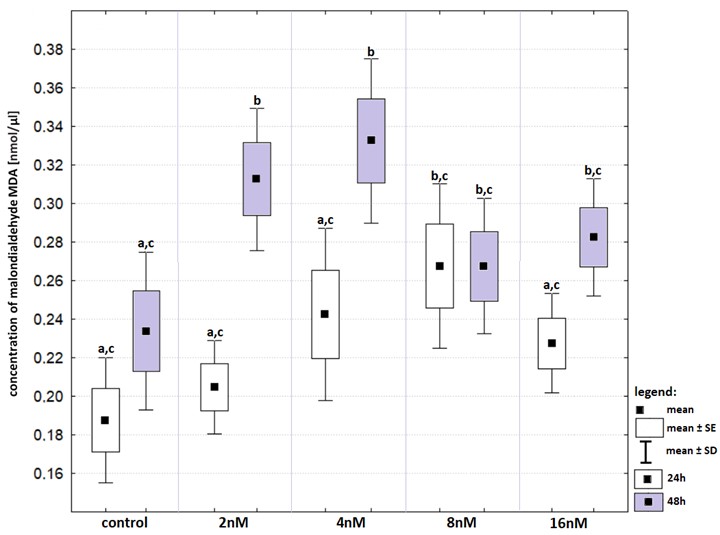

Supplement: Supplemental Information 7 — Values are presented as mean ±SE and mean ±SD. Letters indicate groups that differ significantly from each other (p < 0.05, Kruskal–Wallis test and Dunn’s test as a post hoc); (ac) significantly different from values marked with letter b; (b) significantly different from values marked with letters a and c; (bc) significantly different from values marked with letter a. [file peerj-13-19235-s007.jpg]
